# Ultrasound Mediated Cellular Deflection Results in Cellular Depolarization

**DOI:** 10.1002/advs.202101950

**Published:** 2021-11-07

**Authors:** Aditya Vasan, Jeremy Orosco, Uri Magaram, Marc Duque, Connor Weiss, Yusuf Tufail, Sreekanth H Chalasani, James Friend

**Affiliations:** ^1^ Medically Advanced Devices Laboratory Department of Mechanical and Aerospace Engineering Jacobs School of Engineering and Department of Surgery School of Medicine University of California San Diego La Jolla CA 92093 USA; ^2^ Molecular Neurobiology Laboratory The Salk Institute for Biological Studies La Jolla CA 92037 USA

**Keywords:** acoustofluidics, digital holographic microscopy, neuromodulation, ultrasound

## Abstract

Ultrasound has been used to manipulate cells in both humans and animal models. While intramembrane cavitation and lipid clustering have been suggested as likely mechanisms, they lack experimental evidence. Here, high‐speed digital holographic microscopy (kiloHertz order) is used to visualize the cellular membrane dynamics. It is shown that neuronal and fibroblast membranes deflect about 150 nm upon ultrasound stimulation. Next, a biomechanical model that predicts changes in membrane voltage after ultrasound exposure is developed. Finally, the model predictions are validated using whole‐cell patch clamp electrophysiology on primary neurons. Collectively, it is shown that ultrasound stimulation directly defects the neuronal membrane leading to a change in membrane voltage and subsequent depolarization. The model is consistent with existing data and provides a mechanism for both ultrasound‐evoked neurostimulation and sonogenetic control.

## Graphical abstract

With the help of a new method for imaging cell membrane motion, high‐speed digital holographic microscopy, membrane deformation responsible for the direct depolarization of membranes that lead to action potential generation in nerve cells is observed. A complete model of the phenomena has been devised, producing voltage spiking and oscillation predictions similar to patch clamp measurements.

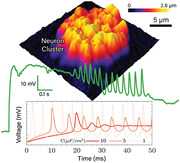

## 1 Introduction

Existing methods to stimulate neural activity include electrical,^[^ ^1^, ^2^, ^3^, ^4^, ^5^ ^]^ optical,^[^ ^6^ ^]^ and chemical techniques.^[^ ^7^ ^]^ They have enabled the development of novel therapies that are used in clinical settings,^[^ ^8^ ^]^ in addition to helping understand aspects of neural function^[^ ^9^ ^]^ and disease mechanisms.^[^ ^10^ ^]^ Despite their beneficial impact, these approaches are fundamentally limited. Electrical stimulation is invasive, requiring direct contact with the target of interest. Inserting electrodes into the brain may lead to inflammation, bleeding, cell death,^[^ ^11^ ^]^ and local cytokine concentration increases in microglia that precipitate astrocyte formation around the electrodes that, in turn, reduce long‐term effectiveness.^[^ ^12^ ^]^ In addition, it may have nonspecific effects depending on the electric field generated by the electrodes and the stimulation parameters used.^[^ ^13^ ^]^ Transcranial direct current stimulation and transcranial magnetic stimulation are new and noninvasive, yet they have poor spatial resolution on the order of 1 cm.^[^ ^14^, ^15^ ^]^ Furthermore, approaches combining genetic tools with light or small molecules achieve cellular specificity. Optogenetics, which involves the use of light and genetically encoded membrane proteins,^[^ ^16^ ^]^ has enabled elucidation of cellular circuits in animal models. However, it remains an invasive technique and applications are limited by the depth of penetration of light in tissue. By contrast, chemogenetics, using small molecule sensitive designer receptors, is limited by poor temporal resolution and is unfortunately impractical for many neural applications that require millisecond response times.^[^ ^17^ ^]^

Ultrasound can overcome the limitations of these methods. It is noninvasive and has a high spatiotemporal resolution (<1 mm and <1 ms) in comparison to existing techniques. Improvements in the spatial resolution through transfection of mechanosensitive proteins currently come at the cost of a minimally‐invasive procedure to directly inject the vector into the target tissue,^[^ ^18^ ^]^ though there may soon be non‐invasive alternatives.^[^ ^19^ ^]^ The spatial resolution of ultrasound is governed by the wavelength of operation and is about 1.5 mm at 1 MHz in tissue. The temporal resolution is dependent on the pulse duration of stimulation and may be as short as a single time period, *T* = 1/*f* where *f* is the operating frequency. The frequency choice is dictated by the depth and size of the target region in traditional focused ultrasound neuromodulation.^[^ ^20^ ^]^ Harvey^[^ ^21^ ^]^ was one of the first to utilize these advantages over 90 years ago on frog ventricular heart tissue. Recent advances in describing the suppression of epileptic activity in patients^[^ ^22^ ^]^ are an indicator the method is still being considered in clinical applications.

Despite these recent experimental and clinical developments, and progress in exploring the sonogenetic and ultrasonic‐to‐chemical action mechanisms, there is no convincing, overarching explanation for the observations reported in vitro or in vivo. Some of the proposed mechanisms include cavitation,^[^ ^23^ ^]^ indirect auditory signaling in vivo^[^ ^24^ ^]^ and increased lipid clustering resulting in a change in the membrane tension.^[^ ^25^ ^]^ These studies have either been conducted on time scales that are orders of magnitude larger than those used for ultrasound neuromodulation, lack robust imaging techniques that operate at timescales relevant to the frequency of stimulation, or use incorrect stimulation thresholds that are orders of magnitude lower than values reported in experimental work.^[^ ^26^ ^]^ Additionally, studies often treat surface tension, membrane composition, and membrane stresses as a single term, membrane fluidity.^[^ ^25^ ^]^ This term lacks rigorous physical description and is assigned a value based on relative fluorescence intensity changes. The imprecision of this description makes it difficult to isolate the influence of the measurable physical mechanisms of which it is comprised. A model using membrane fluidity leaves the explanation of the biophysical phenomenon incomplete.

More broadly, action potentials are known to appear in phase with the cell membrane's deflection.^[^ ^27^, ^28^ ^]^ Pivotal work by Lee et al. ^[^ ^29^ ^]^ investigated neuronal displacement using high‐pressure ultrasound sufficient to induce cavitation believed to be responsible for the observed effects. However, Lee et al.^[^ ^29^ ^]^ acknowledged that it may not play a role in neuromodulation, contrary to models put forth in the past.^[^ ^23^ ^]^ Instead, Lee et al. postulate that neuromodulatory effects may be driven by acoustic radiation forces,^[^ ^30^ ^]^ hinting at the results we later demonstrate in this paper. These and more recent studies into the thermodynamic effects associated with the generation of action potentials^[^ ^31^ ^]^ point to transmembrane voltage changes being more than just an electrical phenomenon, they are possibly influenced by physical motion of the membrane and its components.

All that noted, a key limitation in validating existing models is the inability to measure physical motion across the vast differences in spatiotemporal scales. The ultrasound signal is on the order of 1 MHz and is three orders of magnitude faster than the electrical response of a cell. The wavelength of ultrasound in tissue at these frequencies is orders of magnitude larger than the membrane thickness. Existing methods to measure cell deflection include contact‐based atomic force microscopy,^[^ ^32^, ^33^ ^]^ which has high spatial resolution but poor temporal resolution and lacks the ability to simultaneously scan multiple points.^[^ ^34^ ^]^ Optical tweezers have been used for over twenty years, but only produce results from slow to static deformation of cells and often require attachment of beads or other structures that reduce the measurement to just a few spatial points.^[^ ^35^ ^]^ Traditional digital holographic imaging^[^ ^36^ ^]^ is slow but offers high spatial resolution across a large field of view.

We employ high‐speed digital holographic microscopy (DHM), a unique method established in our group and reported for the first time here. It provides much higher resolution in both space and time than previous methods, and is therefore better suited to the study of dynamics of the cell membrane due to ultrasound. To illustrate this, we provide the first three‐dimensional (3D) visualization of cell membrane deflection due to an ultrasound stimulus using the high‐speed DHM. We use current clamp electrophysiology in the challenging environment of intense ultrasound to monitor ultrasound‐driven, real‐time changes in voltage across the membrane in single neurons in vitro. Furthermore, we have devised an analytical model to predict neuronal depolarization driven by membrane deflection from applied ultrasound stimulus. The experimental results confirm the predictions made by the biophysical model, both with regard to membrane deflection and voltage changes. These findings provide insight into the effects of ultrasound on cells and cell signaling, the understanding of which is vital to sonogenetics and its clinical application.

## 2 Results

### 2.1 Digital Holographic Imaging of Cell Membrane Deflection

**Figure 1:**
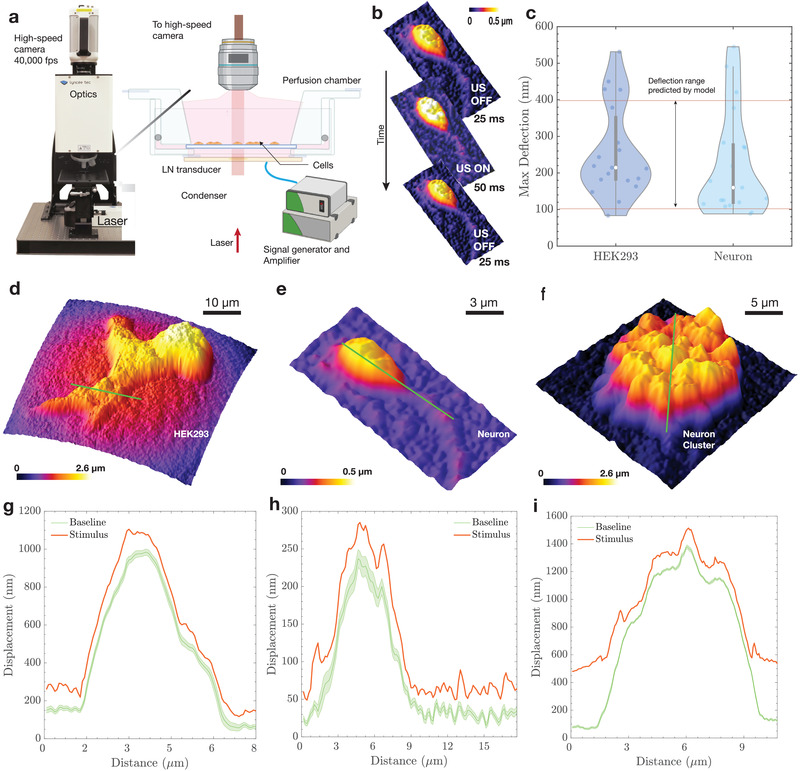
High‐speed DHM imaging of membrane deflection. The deflection of the membrane under the influence of ultrasound was visualized using a) high‐speed digital holographic microscopy (DHM). The DHM setup included a lithium niobate transducer driven by a signal generator and an amplifier at 6.72 MHz. The cells were mounted on a coverslip and placed in a custom perfusion chamber maintained at 37 °C. The DHM enables the b) quantitative reconstruction of phase images acquired by the high‐speed camera at 40 000 frames s^−1^. Each recording began with 25 ms of no stimulus as a baseline, followed by a 50 ms ultrasound stimulus, and ended with a 25 ms baseline. c) The maximum deflection from the mean position was found to be 100–400 nm, with a median deflection of 214 nm for HEK293 cells and 160 nm for neurons (*N* = 30 for each cell type). Reconstructed phase profiles are shown for different cell types: d) HEK293 cells, e) neurons, and f) neuronal clusters. Displacement was measured as a function of distance along the green lines provided in the (d–f) contour plots and were g–i) plotted with (red line plot, max displacement during stimulus) and without (green plot, Baseline) ultrasound stimulus. A distance of “zero” in (g–i) is at the left end of the green line in (d) and (e) and at the bottom of the green line in (f). For the (green) baseline displacement, note the mean and 95% confidence intervals are provided. The maximum variation throughout all baseline responses was less than ±20 nm.

High resolution imaging approaches employing phase‐contrast^[^ ^37^ ^]^ and differential contrast^[^ ^38^ ^]^ are commonly used to image biological specimens. These techniques transform phase differences to amplitude differences in an image, but they lack quantitative phase information. High‐speed DHM^[^ ^39^ ^]^ is a cutting‐edge method that produces 3D holograms at high frame rates. We use transmission DHM, which measures transparent media based on quantifying phase disparities induced by the measured sample. In short, this approach works by comparing phase differences induced in the coherent light transmitted through the sample with reference light traversing an unobstructed path. Digital holographic microscopy has several advantages in comparison to conventional microscopic techniques. Numerical processing of the wavefront transmitted through the sample permits simultaneous computation of intensity and phase distribution.^[^ ^40^ ^]^ The holographic measurements also make it possible to focus on different object planes without relative movement between the stage and the lens^[^ ^41^ ^]^ and enables numerical lens aberration correction.^[^ ^42^ ^]^ Our unique DHM system operates at high frame rates (40 000 frames s^−1^) and consists of a custom‐built perfusion chamber with a built‐in ultrasound transducer (**Figure** **1**a). A heated stage keeps the media at a constant temperature over the duration of the recording. The system reconstructs phase images of cells that are then analyzed to determine the baseline profile (prior to ultrasound), during exposure to ultrasound, and afterward. This enables us to accurately visualize the maximum displacement of the membrane from the mean position under the influence of ultrasound.

The measurements of apical cellular membrane deflection due to ultrasound consisted of a 25 ms baseline recording, followed by a 50 ms ultrasound stimulus and a 25 ms post‐stimulus dwell (Figure 1b), leading to a median deflection of 214 nm for human embryonic kidney (HEK293) cells and 159 nm for neurons, with a range of 100 to 550 nm across the two tested cell types (Figure 1c and Videos S1 and S2, Supporting Information). These stimulation parameters are consistent with prior studies for calcium imaging in vitro and in vivo^[^ ^18^ ^]^ and are consistent with recommendations from past important work.^[^ ^43^ ^]^ Sample reconstructed phase images of HEK293 cells, neurons and neuronal clusters are shown in Figure 1d–f. The baseline deflection for these samples, including a 95% confidence interval, had a range of ± 20 nm, inclusive of both random thermal fluctuations across the cell membrane and potential noise introduced to the system due to the imaging arrangement (Figure 1g–i). Sample displacement baseline membrane profiles are illustrated in Figure 1g–h (see Videos S1 and S2, Supporting Information as well) for HEK293 cells and neurons, and Figure 1i represents the deflection profile for a cluster of neurons. The cluster was imaged to confirm deflection in a group of neurons and help provide insight into the in vivo mechanisms of activation. Results from the neuronal cluster show that the magnitude of deflection remains roughly the same for a group of cells as for a single neuron. The larger deflection at the edges of the cluster is due to the neurons at the edges being less constrained in comparison to the ones in the center.

Membrane deflection during the generation of action potentials has been observed in the past,^[^ ^44^ ^]^ but the converse phenomenon of membrane deflection leading to the generation of action potentials has not been explored at the level of an individual neuron using clinically relevant stimulation pressures. As described before, other imaging techniques have been reported for measuring cell membrane deflection, but are unable to match the spatiotemporal capabilities of the high‐speed DHM technique. Overall, our experimental setup allows us to confirm membrane deflection due to ultrasound for cells adherent to a coverslip and we relate these results to a mathematical model in the following section.

### 2.2 Membrane Deflection Model

Based upon the results from the experiments, with cells cultured on a surface and surrounded by media, the membrane is assumed to be fixed at the periphery. A similar case occurs in vivo, where the extracellular matrix holds individual cells in place and provides anchoring locations for sections of the membrane.^[^ ^45^ ^]^ Cellular anchoring is important because it imposes a characteristic distance over which the range of permissible deflection wavemodes may occur (see Experimental Section). Its deflection is restricted in the analysis to a single direction, perpendicular to the plane of the membrane and parallel to the direction of propagation of sound. The model does not take into account the restoring effects of the actin cytoskeleton, which is difficult to estimate and likely plays an important role in restoring the membrane to its original equilibrium position.

The stimulus provided to the cells is in the form of a sinusoidal burst, a short‐term continuously oscillating ultrasound signal of constant amplitude and frequency. In a burst, a sinusoidal electrical signal is typically applied across the piezoelectric material used in a transducer, which transforms this signal into a sinusoidally varying pressure field in the fluid medium at the frequency of excitation. This is rather different than the approach used by Prieto et. al,^[^ ^26^ ^]^ where the ultrasound is modeled as a step increase in hydrostatic pressure from zero to a fixed positive value at *t* = 0. In our approach (see Experimental Section), the burst signal oscillates at the ultrasound frequency, and an analytical solution for the slower time scale of the membrane mechanics is found in response to this harmonic ultrasound excitation. This solution is then used in a numerical model to produce the solution for the deflection of the fixed membrane, resolving the discrepancy between the timescales of ultrasonic stimulation (≈0.1 μs) and the experimentally verified membrane deflection occurring on the order of milliseconds. This hybrid approach was chosen because a numerical simulation of the entire phenomena from ultrasound to membrane deflection would be extremely difficult due to the vastly different spatiotemporal scales, even with state‐of‐the‐art computational resources. Finally, the hydrostatic pressure included by Prieto et. al^[^ ^26^ ^]^ is discarded here, because it is orders of magnitude lower than the ultrasonic radiation pressure.

The damped wave equation describing the deflection, *u*, of the membrane in response to ultrasonic pressure, *P*_US_, is written as

(1) $$\rho \;\partial_{t}^{2}\;u=2\;\eta \;\frac{\partial^{3}u}{\partial x^{2}\partial t}+\left(2\;\gamma \;\partial_{x}^{2}u+P_{\text{US}}\right)\;\left(\frac{\pi }{d}\right)$$

where ρ and η are the dynamic viscosity and density of the surrounding fluid, both assumed to be the same as water as used in prior studies^[^ ^46^, ^47^ ^]^); γ is the surface tension between the membrane and media; and *d* is the characteristic length of the membrane between anchor points. Equation (1) was solved by the method of eigenfunction expansion (see Experimental Section). Figure 1 provides results representative of the analysis, with a 1 MPa pressure supplied to the membrane using a 7 MHz transducer in the form of a sine wave over a period of 5 ms. The mechanical index for the parameters listed in this study is 0.37, well below the oft‐cited mechanical index threshold for cavitation onset of 0.7 in bubble‐perfused tissue.^[^ ^48^ ^]^ However, our study uses no bubbles. In this case, the U.S. Federal Drug Administration's mandated clinical safety threshold index of 1.9 without introduced microbubbles^[^ ^49^, ^50^ ^]^ is more appropriate. These data suggest that we are unlikely to cause cavitation and cell viability remains unaffected as shown by prior work with similar stimulus parameters.^[^ ^18^ ^]^

**Figure 2:**
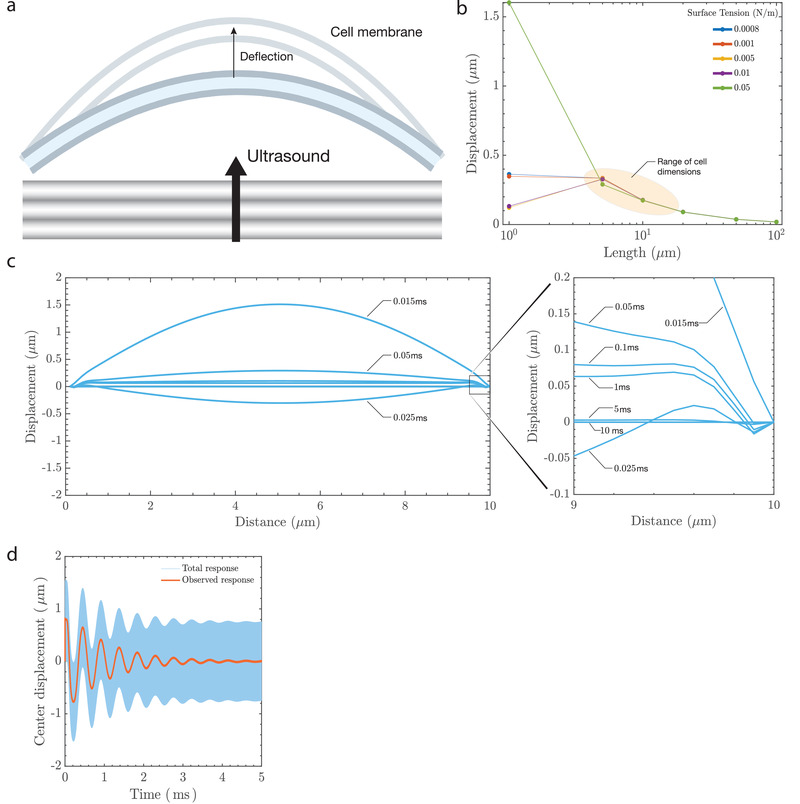
Prediction of membrane deflection due to ultrasound. Ultrasound results in a) membrane deflection that triggers a transmembrane electrical response. The cell membrane bilayer stretches, increasing its area, and the outer leaflet of the bilayer will likely deflect more than the inner leaflet due to the presence of cytoskeletal components such as actin and microtubules that anchor the inner leaflet. Two of the factors that affect membrane displacement are surface tension of the lipid membrane and the length under consideration. The model b) predicts displacements between 100–400 nm for dimensions that correspond to the size of a cell (5–20 μm) and is within the limits observed using the DHM. The response is c) dynamic, with snapshots of the predicted deflection at different times (in ms) across a 10 μm wide membrane section that is anchored at the ends. The maximum deflection occurs when the stimulus is first provided and there is a balance between viscous dissipation and conservative effects of inertia and surface tension (see “Sustaining Oscillations on the Membrane” in Experimental Section and Section 2.2) which lead to sustained wavemodes on the membrane at the millisecond timescale (observed response). A low‐pass temporal filter of the membrane's center displacement at 5 μm indicates d) an oscillatory deflection over the stimulus duration of 5 ms.

Maximum membrane deflection occurs when the ultrasound stimulus is applied (**Figure** **2**a), followed by decay due to viscous losses to the host medium. The magnitude of deflection depends on the stimulation frequency and peak pressure, with lower frequencies and higher pressures producing greater membrane deflection. The critical parameters that influence the deflection magnitude are the characteristic membrane anchor length and surface tension, as shown in Figure 2b. The deflection predicted by the model for dimensions relevant to the size of a cell are between 100 to 400 nm, irrespective of the value of surface tension for an anchor length ranging from 5–20 μm based on the average size of the soma^[^ ^51^ ^]^ and average diameter of HEK293 cells.^[^ ^52^ ^]^ We modeled membrane deflection due to a range of surface tension values reported in the literature.^[^ ^26^, ^53^ ^]^ Maximum membrane deflection occurs at the midpoint of the axisymmetric membrane model. This is portrayed in Figure 2c, where we provide graphical “snapshots” of the ultrasonically‐forced membrane over time. The closed‐form displacement solution to Equation (1) allows us to link the fast ultrasonic timescales (on μs order, or, total response) to phenomena occurring at observable timescales (on ms order, or, observed response), as shown in Figure 2d. The character of the membrane “slow time” response—that is, its ability (or lack thereof) to sustain oscillations—is governed by the value of the Ohnesorge number, *Oh*. The term is defined in this way because the membrane oscillations typically occur slowly: at a frequency far less than the incident ultrasound.

The nondimensional parameter *Oh* characterizes the importance of dissipative viscous forces relative to the combined interaction of conservative inertial and surface tension forces. In other words, *Oh* characterizes, on average, the extent to which the membrane dissipates or conserves mechanical energy. Typical *Oh* values for neurons range from ≈0.06 to ≈0.45 based on values of surface tension, viscosity, and membrane length considered in this work. This implies that inertial and surface tension forces dominate over viscous forces: the slow time membrane response is characteristically oscillatory. This behavior results from the membrane's tendency toward retaining mechanical energy in the form of sustained oscillations when $Oh<\sqrt{2/\pi }\approx 0.8$. This is explicitly derived in the detailed analysis (see Experimental Section) and suggests that the slow time oscillations of the ultrasonically actuated membrane is implicated in the changes in the membrane capacitance as detailed in the following sections.

### 2.3 Model Prediction of Action Potentials and Electrophysiology

To model the electrical output of a neuron under the influence of ultrasound, a modified version of the original Hodgkin–Huxley equations is first used^[^ ^54^ ^]^

(2) $$\frac{dV_{m}}{dt}=-\frac{1}{C_{m}}\left[I_{\text{app}}+I_{\text{Na}}+I_{\text{Kd}}+I_{M}+I_{\text{leak}}\right]$$

In this equation, the membrane potential of the neuron, *V*_m_, changes over time with respect to the membrane capacitance, *C*_m_, and the underlying currents, *I*_app_, *I*_Na_, *I*_Kd_, *I*_M_, and *I*_leak_. At rest, *V*_m_ = −71.9 mV is the well‐known membrane potential of the cell and, notably, the action potential generation is controlled by the presence of an applied current, *I*_app_, while the other currents are based on the membrane morphology and chemistry and are detailed in Experimental Section. The increase of *I*_app_ beyond a certain threshold produces spiking behavior typical of neurons.

**Figure 3:**
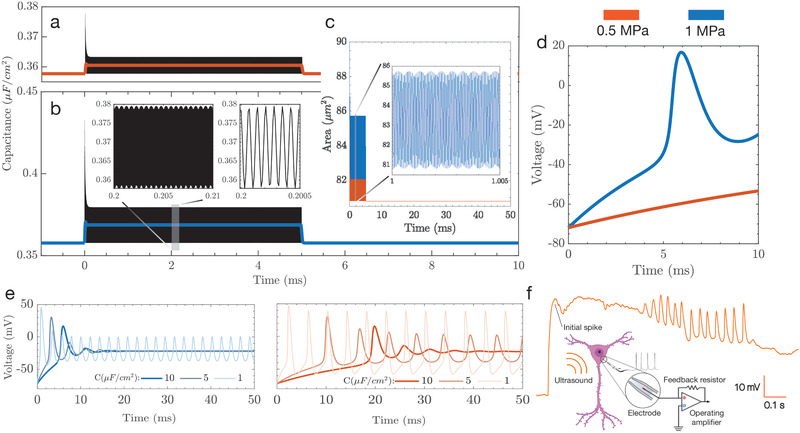
Displacement driven capacitance changes result in action potential generation. a–e) Simulations help inform the development of stimulus parameters, in terms of time and pressure amplitude; note that throughout (a–e) 0.5 MPa stimulation is red while 1 MPa is blue. The capacitance changes are plotted over the stimulus duration (5 ms) for a) 0.5 and b) 1 MPa with the corresponding area changes that cause c) capacitance fluctuations. The capacitance fluctuations produce depolarization at 1 MPa, but not at 0.5 MPa, indicating d) the presence of a pressure threshold to stimulate neurons. e) Over a longer 50 ms stimulus, the action potential evolves quite differently over time for the two acoustic pressures. At lower pressures, longer stimuli may be necessary to produce action potentials. f) In vitro current clamp electrophysiology was used to verify the predictions of the model and shows that the presence of a preliminary spike followed by oscillations in voltage across the membrane.

The capacitance, *C*_m_, may also fluctuate due to a morphological change in the membrane. Such a modification is not modeled in the original representation of this equation, but it may be included. The voltage change as described in Equation (2) includes a time‐dependent capacitive current, $I_{\text{app}}\equiv V_{m}\frac{dC_{m}}{dt}$. With this included in Equation (2), it is possible to solve the differential equation for the voltage and gating variables while incorporating the capacitance change due to membrane deflection. Membrane deflection is constrained to a certain extent due to parts of the cell that are adherent to the substrate or the extracellular matrix. This causes an increase in area between the adherent locations and with sufficient deflection, this produces a depolarization across the membrane. The value of the transmembrane voltage is dependent on the magnitude and duration of the applied stimulus. **Figure** **3** indicates the change in capacitance due to 6.72 MHz ultrasound at 0.5 (Figure 3a) and 1 MPa (Figure 3b) with the corresponding area fluctuations that bring about the change in capacitance represented in Figure 3c. In order to compute the time‐dependent membrane area variation, we extract the slow time output of Equation (1) for use with the axisymmetric area integral. The capacitance of the membrane is then determined by treating it as a dielectric between charged surfaces. This produces a slow time capacitive response, bearing an order of magnitude equivalence to the ion channel relaxation times in the modified Hodgkin–Huxley model.^[^ ^55^ ^]^

The stimulus of 1 MPa results in depolarization as indicated in Figure 3d, while the lower pressure does not result in the generation of an action potential over the stimulus duration. Reported values of baseline membrane capacitance have been shown to vary,^[^ ^56^ ^]^ and we show that longer stimuli will result in the generation of action potentials as a cumulative effect of capacitance change over the duration of the stimulus. Figure 3e represents transmembrane voltage changes for a stimulus of 50 ms. We notice that depolarization takes place in both cases. However, initial spikes are delayed by up to 20 ms in the lower pressure case, indicating the need for increased stimulus durations for lower pressures. Our model also shows a lower spike frequency for the 0.5 MPa case in comparison to 1 MPa. The simulation output of our model for the lower pressure and longer stimulus duration case were verified experimentally using voltage clamp electrophysiology (Figure 3f) and shows an initial spike corresponding to the delivery of the ultrasound stimulus, followed by oscillations.

## 3 Discussion

We model how ultrasound results in membrane deflection and eventually leads to transmembrane voltage changes. In a first, we demonstrate real‐time membrane deflection due to ultrasound using high‐speed DHM imaging (Videos S1 and S2, Supporting Information). We leverage the Hodgkin–Huxley equations, which are a set of phenomenological equations describing action potential generation in a squid axon and are one of the most important neuronal models. However, observations of mechanical deflection accompanying action potentials^[^ ^44^ ^]^ show that the underlying assumptions of the Hodgkin–Huxley model may need to be revisited, as there are mechanical phenomena involved. In the context of ultrasound neuromodulation, our model presents insights into the generation of action potentials due to mechanical deflections and is theoretically supported by models such as the ones put forth in the past few years.^[^ ^31^, ^57^ ^]^ The deflection due to the applied ultrasound stimulus results in a net area change of the membrane between the two pin locations that represent an adherent cell. The area changes take place elastically while maintaining constant volume. This results in a change in capacitance that, when incorporated in the Hodgkin–Huxley model, results in transmembrane voltage changes. Capacitance of the membrane can be modeled using an expression for a parallel plate capacitor,^[^ ^58^ ^]^ and an increase in area results in a proportional increase in capacitance (see Experimental Section).

The model does not take into account restoring effects of the actin cytoskeleton, whose influence will lower the membrane deflection and cause the inner leaflet to deflect less than the outer leaflet. However, this cannot account for the ≈100 nm deflection experimentally observed in this work, and only plays a minor role in bringing about capacitance changes according to previous studies.^[^ ^23^ ^]^ The model and the use of high‐speed DHM imaging present opportunities for exploring the influence of ultrasound on native neurons and HEK293 cells, as presented here. A combination of fluorescence imaging with DHM can be used to image focal adhesions and cells that have been engineered to express membrane proteins that are sensitive to ultrasound stimuli, in other words using sonogenetics.^[^ ^59^ ^]^ At a cellular level, there are two proposed models for the activation of a mechanically‐gated ion channel: the force from lipid model and the force from filament model. The force from lipid model was put forth by Martinac et al.^[^ ^60^ ^]^ and proposes that changes in membrane tension or local membrane curvature result in opening or closing of channels. In the force from filament model,^[^ ^53^ ^]^ the stimulus is transferred to tethers that connect the membrane to the cytoskeleton. Conformational changes in the tethers result in opening or closing of the channel. In reality, both models play a part in opening and closing a given channel.

Although it is difficult to estimate the relative contribution of these mechanisms, it is possible to estimate the deflection of the cell membrane as highlighted in the preceding sections. This is of particular significance when we consider the membrane‐bound proteins such as TRPA1, MsCL,^[^ ^61^ ^]^ Piezo,^[^ ^62^ ^]^ and their interaction with the actin network. Disruption of the actin cytoskeleton has been shown to reduce mechanosensitive activity of such ion channels^[^ ^63^ ^]^ and it is possibly due to decreased separation between the leaflets of the bilayer when the actin network is disrupted. In addition to quantifying the deflection due to mechanosensitive proteins, there is potential to quantify the forces on the cell due to ultrasound using Förster resonance energy transfer force sensors.^[^ ^64^ ^]^

Our model also predicts the generation of action potentials from capacitive changes that occur when the adherent cell is exposed to ultrasound. Charge across the membrane is maintained by a gradient in ion concentration across the cell membrane, with Na^+^ ions on the outside and Cl^−^ ions on the inside, resulting in a net negative resting potential. As the membrane deflects, it is partially constrained by the adherent regions, resulting in an increase in area of the membrane between the adherent locations. An increase in the area of the membrane directly increases its capacitance (see Equation (18) in Experimental Section). This relationship between area, capacitance, and transmembrane voltage change has also been indicated in prior publications that investigate, outside the context of ultrasound neuromodulation, the capacitive properties of biological membranes.^[^ ^58^ ^]^

We demonstrate transmembrane voltage changes for two cases, a pressure of 0.5 and 1 MPa and observe that voltage changes only take place for the higher pressure case for lower stimulus durations, defining a pressure threshold dependent upon the duration of stimulus. We also investigate the influence of longer stimulus durations on the generation of action potentials for different values of baseline capacitance. As verified by a current clamp electrophysiology study in the whole cell configuration, increased stimulus durations even at lower pressures result in action potential generation, though with lower spike rates. The parameters used in this study are similar to prior work in vivo^[^ ^18^ ^]^ and deflection has been shown by other groups to occur in vivo by Lee et al.,^[^ ^29^ ^]^ although at much higher pressures and with cavitation.

One of the limitations with performing single cell current clamp electrophysiology while using ultrasound at amplitudes sufficient to drive a physiological response is the loss of a seal between the membrane and the patch pipette due to the membrane's deflection. There are, however, reports of current clamp electrophysiology results with ultrasound using microbubbles^[^ ^65^ ^]^ and at much higher frequencies^[^ ^66^ ^]^ or with devices.^[^ ^67^ ^]^ In each of these three cases, there is reason to believe that while the stimulation techniques or device may work for in vitro work, they will not be suitable for in vivo work. One potential way to overcome this issue would be to perform electrophysiological recordings for cells encased in matrigel that would limit the movement of the recording pipette with respect to the membrane.

Until now, the mechanisms underlying ultrasound neuromodulation have lacked explanation and existing models lack experimental data. Taken together, our results offer valuable insight into the underlying effects of ultrasound on cell membranes, as well as insight into how these effects translate to transmembrane voltage changes. The predictions of our model were confirmed using a novel, high‐speed imaging technique. We were able to visualize and quantify membrane deflection in real‐time and predict depolarization due to the imposed ultrasound stimulus.

## 4 Experimental Section

### HEK293 Cell Culture

Human embryonic kidney (HEK293) cells (CRL‐1573, ATCC, Manassas, Virginia, USA) were cultured using standard procedure in DMEM supplemented with 10% fetal bovine serum and 20 mm glutamine in a 37~°C and 5% CO_2_ incubator. Cells beyond passage 30 were discarded and a new aliquot was thawed. For experimental plating, 18 mm coverslips were coated with poly‐d‐lysine (PDL; 10 g L^−1^, minimum 2 h, P6407, Sigma‐Aldrich, St. Louis, Missouri, USA), and HEK293 cells were seeded at 150K, 200K, or 250K cells mL^−1^ for 24 h before the experiment. Cells were allowed to grow over 24 h and a balance was struck between an increase in the cell density to improve cell health and the need to perform observations with the DHM that improved as the cell density was decreased. The cells were healthier at a higher density, but the DHM relied on contrast between a given cell and its environment, which was reduced as the cell density increases. For imaging, coverslips were mounted on a specialized chamber featuring an ultrasound transducer ≈2 mm below the coverslip and a 10 mL reservoir of media above the coverslip. Once cells were in focus, a 6.72 MHz ultrasound pulse of 50 ms duration was delivered while imaging with an immersion objective as described in following sections, and a cell membrane profile was reconstructed and analyzed.

### High‐Speed Digital Holographic Microscopy

HEK293 cells and neurons were observed through a 40×, 0.8 NA (numerical aperture) water immersion microscope objective. The field of view used for the setup was 60.5 μm × 60.5 μm, with a vertical accuracy and repeatability of 4 and 0.08 nm respectively.^[^ ^68^ ^]^ Holograms were recorded using a high‐speed camera (Nova S12, Photron, San Diego, California, USA). Acquisition and reconstruction were performed using custom software (Koala, Lynceé‐tec Inc., Lausanne, Switzerland) on a computer workstation. Data were recorded on a separate computer equipped with a solid‐state drive, with each 100 ms recording equating to ≈20 GB of data. The observations reported in this study represent a combined analysis of 1.4 TB of data. The data were reconstructed after each batch of six coverslips was processed in order to reduce the time between trials and to ensure optimum cell health. The setup consisted of a custom perfusion chamber that was built to accommodate a lithium niobate transducer operating at 6.72 MHz. The perfusion chamber was housed on a stage maintained at 37 °C (Figure 2a) using a heated stage (Bioscience Tools TC‐100s).

### Modeling of Deflection and Transmembrane Voltage Changes

As the pressure wave propagated through the fluid and contacted the adherent cell, the region of the cell membrane between adhesion zones deflected. This deflection led to a change in area of the membrane and causes a capacitance change. The two‐dimensional model assumed that the membrane had a known value of surface tension.^[^ ^69^ ^]^ The membrane was surrounded by a fluid, assumed to have the properties of water in this case. The vertical displacement of the membrane was approximated to be equal to the displacement of the fluid just above the membrane. The start was with a simplified version of the Navier–Stokes equation

(3) $$\begin{array}{c} \rho \;\left(\partial_{t}\;v+v\cdot \nabla \;v\right)=\eta \;\nabla^{2}\;v-\nabla \;P \end{array}$$

where ρ and η are the density and viscosity of water, respectively. The expression ∇*P* is the pressure gradient and *v* is the velocity. In Equation (3), the convective acceleration is *v* · ∇*v* = 0 as the flow is unidirectional in *z* ^[^ ^70^ ^]^ and the fluid is assumed to be incompressible. The membrane was symmetric in *x* and *y*, allowing the viscous term to be simplified as ∂_*x*_ *v*_*z*_ = ∂_*y*_ *v*_*z*_. What was left were

(4) $$\begin{array}{c} \rho \;\partial_{t}\;v_{z}=2\;\eta \;\partial_{x}^{2}\;v_{z}-\nabla \;P \end{array}$$

The net pressure gradient in this case is a function of the time dependent pressure in the fluid due to ultrasound and the surface tension of the membrane, which resists deformation

(5) $$\begin{array}{c} \nabla \;P=-\;(\;2\;\gamma \;\partial_{x}^{2}\;u+P_{\text{US}})\;\pi \;d \end{array}$$

where *u* is the displacement in *z* and *P*_US_ is the pressure due to an ultrasound source, typically acting in the form of a sinusoidal pulse, *P*_US_ = *P*_0_sin (ω*t*), where ω = 2π*f*. By contrast, Prieto et al.^[^ ^26^ ^]^ at this point chose to represent the ultrasound as a step change in the pressure, from a static, zero relative pressure to a static positive value at time *t* = 0 well below the pressure amplitudes used in experimental studies, typically 1 kPa to 1 MPa. Prieto et al.'s representation was numerically attractive but difficult to reconcile with the harmonic oscillatory pressure delivered by the transducer. In the absence of an analytical solution for the ultrasound propagating through the medium and membrane, one would be forced to numerically represent the MHz‐order sinusoidal signal with sufficiently small spatiotemporal step sizes to satisfy the Nyquist criterion, and do so for at least several hundred milliseconds to determine the response of the cell membrane to the ultrasound pressure oscillation, producing very large models with many millions to billions of temporal steps for a single solution. Consequently, these past studies were understandably forced to make spurious approximations^[^ ^71^ ^]^ to avoid impossibly prohibitive computation times.

Substituting this into Equation (4) produced a partial differential equation for the displacement of the membrane driven by ultrasound

(6) $$\begin{array}{c} \rho \;\partial_{t}^{2}\;u=2\;\eta \;\frac{\partial^{3}u}{\partial x^{2}\partial t}+\left(2\;\gamma \;\partial_{x}^{2}u+P_{\text{US}}\right)\;\left(\frac{\pi }{d}\right) \end{array}$$

The boundary conditions are the clamped conditions at the ends of the membrane and the initial displacement condition

(7a) $$\begin{array}{cc} u(0,t) & =0 \end{array}$$

(7b) $$\begin{array}{cc} u(d,t) & =0 \end{array}$$

(7c) $$\begin{array}{cc} u(x,0) & =\frac{P_{0}\;x\;\left(d-x\right)}{4\;\gamma }\equiv u_{0}\left(x\right) \end{array}$$

(7d) $$\begin{array}{cc} \partial_{t}\;u\left(x,0\right) & =0 \end{array}$$

If hydrostatic pressure is included, the initial condition for membrane displacement may be found by solving $P_{0}+2\;\gamma \;\partial_{x}^{2}\;u=0$. The general solution to partial differential Equation (6) was obtained with the method of eigenfunction expansion, as outlined further on. This was achieved using an orthogonal eigenbasis

(8) $$\begin{array}{c} \phi_{n}\left(x\right)=\sin\left(\sqrt{\chi_{n}}\;x\right) \end{array}$$

where χ_*n*_ = (*n* π/*d*)^2^ corresponds to the *n*th wavemode for a membrane with diameter *d*. Expanding *u* gives

(9) $$\begin{array}{c} u\left(x,t\right)=\sum_{n}\;u_{n}\left(x,t\right)=\sum_{n}\;h_{n}\left(t\right)\;\phi_{n}\left(x\right) \end{array}$$

so that clearly the even modes vanish and *n* = 2 *k* + 1 may be written, and k∈Z≥0 where Z is an integer set. Substituting this expression into Equation (6), one has

(10) $$\begin{array}{c} \sum_{n}\left({\ddot{h}}_{n}+c_{1}\;\chi_{n}\;{\dot{h}}_{n}+c_{0}\;\chi_{n}\;h_{n}\right)\;\phi_{n}\left(x\right)=f\left(t\right) \end{array}$$

where *c*_1_ = 2 η/ρ and *c*_0_ = 2 π γ/ρ *d*, are written in terms of the density of the surrounding fluid, ρ; the viscosity of the surrounding fluid, η; the surface tension along the fluid‐membrane interface, γ; and the membrane diameter, *d*. By multiplying both sides by ϕ_*m*_(*x*) (with $m\in Z^{+}$), integrating over *x* from 0 to *d*, and then leveraging the orthogonality of sines, it was found that the time‐dependent component for the *n*th eigenmode satisfied the second‐order ordinary differential equation

(11) $$\begin{array}{c} {\ddot{h}}_{n}+b_{1,n}\;{\dot{h}}_{n}+b_{0,n}\;h_{n}={\hat{f}}_{n}\left(t\right) \end{array}$$

where *b*_1, *n*_ = *c*_1_ χ_*n*_, *b*_0, *n*_ = *c*_0_ χ_*n*_, and

(12) $$\begin{array}{c} {\hat{f}}_{n}\left(t\right)=\frac{2}{d}\int_{0}^{d}\phi_{n}\left(x\right)\;f\left(t\right)\;dx=\frac{2\;(1-{\left(-1\right)}^{n})}{n\;\pi }\;f\left(t\right) \end{array}$$

The means for obtaining a solution to equations of the form Equation (11) is well known. The homogeneous solution and its coefficients are given by

(13) $$\begin{array}{c} h_{n}^{\left(h\right)}\left(t\right)=a_{+,n}^{\left(h\right)}\;e^{r_{+,n}\;t}+a_{-,n}^{\left(h\right)}\;e^{r_{-,n}\;t} \end{array}$$

where the coefficients $a_{+,n}^{\left(h\right)}$ and $a_{-,n}^{\left(h\right)}$ are

(14a) $$\begin{array}{c} a_{+,n}^{\left(h\right)}=\frac{r_{-,n}}{r_{-,n}-r_{+,n}}\;h_{n}\left(0\right) \end{array}$$

(14b) $$\begin{array}{c} a_{-,n}^{\left(h\right)}=\frac{r_{+,n}}{r_{+,n}-r_{-,n}}\;h_{n}\left(0\right) \end{array}$$

The inhomogeneous solution is

(15) $$\begin{array}{c} h_{n}^{\left(i\right)}\left(t\right)=\frac{1}{r_{+,n}-r_{-,n}}\left(e^{r_{-,n}\;t}\;I_{-,n}\left(t\right)-e^{r_{+,n}\;t}\;I_{+,n}\left(t\right)\right) \end{array}$$

where

(16) $$\begin{array}{c} I_{\pm ,n}\left(t\right)=\int_{0}^{t}e^{-r_{\pm ,n}\tau }\hat{f}\left(\tau \right)\;d\tau \end{array}$$

The total waveform solution was then numerically implemented by taking a finite‐term approximation of Equation (9).

The change in area, *A*, of the membrane then be calculated once the time‐dependent membrane deflection is obtained

(17) $$\begin{array}{c} A=\int_{0}^{d}\;2\pi \;\sqrt{\left(1+{\left(\partial_{x}u\right)}^{2}\right)}\;dx \end{array}$$

By extension, this allowed to determine the change in membrane capacitance, *C*, due to the area change

(18) $$\begin{array}{c} C=\frac{\varepsilon_{0}\varepsilon A}{L} \end{array}$$

where it was regarded that the membrane was a dielectric between two charged surfaces. In this case, *L* is the thickness of the bilayer and has values between 4 and 9 nm, and the relative permittivity, ϵ, has a value of 2.^[^ ^72^ ^]^

The above value of capacitance change was coupled with the modified Hodgkin–Huxley neuronal model, where the capacitive current is defined as $I_{\text{app}}\equiv V_{m}\frac{dC_{m}}{dt}$. This model contained a voltage‐gated sodium current and delayed‐rectifier potassium current to generate actions, a slow non‐inactivating potassium current to recapitulate the spike‐frequency adaptation behavior seen in thalamocortical cells, and a leakage current.

Equation (19) defines the voltage‐gated Na^+^ current where ${\bar{g}}_{\text{Na}}=56$mS cm^−^^2^ is the maximal conductance and *E*_Na_ = 50 mV is the Nernst potential of the Na^+^ channels. The parameter *V*_th_ = −56.2 mV sets the spike threshold

(19) $$I_{\text{Na}}={\bar{g}}_{\text{Na}}\cdot m^{3}\cdot h\cdot \left(V_{m}-E_{\text{Na}}\right)$$

where the gating variables *m* and *h* vary with time according to

(20a) $$\begin{array}{cc} \frac{dm}{dt} & =\alpha_{m}\cdot \left(1-m\right)-\beta_{m}\cdot m \end{array}$$

(20b) $$\begin{array}{cc} \frac{dh}{dt} & =\alpha_{h}\cdot \left(1-h\right)-\beta_{h}\cdot h \end{array}$$

(20c) $$\begin{array}{cc} \alpha_{m} & =\frac{-0.32\cdot \left(V_{m}-V_{\text{th}}-13\right)}{\exp\left[-(V_{m}-V_{\text{th}}-13)/4\right]-1} \end{array}$$

(20d) $$\begin{array}{cc} \beta_{m} & =\frac{0.28\cdot \left(V_{m}-V_{\text{th}}-40\right)}{\exp\left[(V_{m}-V_{\text{th}}-40)/5\right]-1} \end{array}$$

(20e) $$\begin{array}{cc} \alpha_{h} & =0.128\cdot \exp\left[-(V_{m}-V_{\text{th}}-17)/18\right] \end{array}$$

(20f) $$\begin{array}{cc} \beta_{h} & =\frac{4}{1+\exp\left[-(V_{m}-V_{\text{th}}-40)/5\right]}. \end{array}$$

The delayed rectifier K^+^ current is

(21) $$I_{\text{Kd}}={\bar{g}}_{\text{Kd}}\cdot n^{4}\cdot \left(V_{m}-E_{K}\right)$$

where ${\bar{g}}_{\text{Kd}}=6$mS cm^−2^ is the maximal conductance of the delayed‐rectifier K^+^ channels and *E*_K_ = −90 mV is the Nernst potential of the K^+^ channels, and with *n* evolving over time as

(22a) $$\begin{array}{cc} \frac{dn}{dt} & =\alpha_{n}\cdot \left(1-n\right)-\beta_{n}\cdot n \end{array}$$

(22b) $$\begin{array}{cc} \alpha_{n} & =\frac{-0.032\cdot \left(V_{m}-V_{\text{th}}-15\right)}{\exp\left[-(V_{m}-V_{\text{th}}-15)/5\right]-1} \end{array}$$

(22c) $$\begin{array}{cc} \beta_{n} & =0.5\cdot \exp\left[-(V_{m}-V_{\text{th}}-10)/40\right] \end{array}$$

A slow non‐inactivating K^+^ current may be defined as

(23) $$I_{M}={\bar{g}}_{M}\cdot p\cdot \left(V_{m}-E_{K}\right)$$

where ${\bar{g}}_{M}=0.075$mS cm^−2^ is the maximal conductance and τ_max_ = 608 ms is the decay time constant for adaptation of the slow noninactivation K^+^ channels. The parameter *p* is such that

(24a) $$\begin{array}{cc} \frac{dp}{dt} & =\frac{p_{\infty }-p}{\tau_{p}} \end{array}$$

(24b) $$\begin{array}{cc} p_{\infty } & =\frac{1}{1+\exp\left[-(V_{m}+35)/10\right]} \end{array}$$

(24c) $$\begin{array}{cc} \tau_{p} & =\frac{\tau_{\max}}{3.3\cdot \exp\left[(V_{m}+35)/20\right]+\exp\left[-(V_{m}+35)/20\right]} \end{array}$$

The leakage current is

(25) $$I_{\text{Leak}}={\bar{g}}_{\text{Leak}}\cdot \left(V_{m}-E_{\text{Leak}}\right)$$

where ${\bar{g}}_{\text{Leak}}=0.0205$mS cm^−2^ is the maximal conductance and *E*_Leak_ = −70.3 mV is the Nernst potential of the non‐voltage‐dependent, nonspecific ion channels.

The following initial conditions were set for the gating terms

(26a) $$\begin{array}{cc} m_{0} & =\frac{\alpha_{m}}{\alpha_{m}+\beta_{m}} \end{array}$$

(26b) $$\begin{array}{cc} h_{0} & =\frac{\alpha_{h}}{\alpha_{h}+\beta_{h}} \end{array}$$

(26c) $$\begin{array}{cc} n_{0} & =\frac{\alpha_{n}}{\alpha_{n}+\beta_{n}} \end{array}$$

(26d) $$\begin{array}{cc} p_{0} & =p_{\infty } \end{array}$$

Equations (19)–(24) were solved with initial conditions (26) to obtain the transmembrane voltage change of a neuron when subjected to ultrasound stimuli.

### Sustaining Oscillations on the Membrane

A better understanding of the membrane wave propagation can be obtained by considering the decay transience of the constituent wavemodes within the context of the solution to Equation (11). Each wavemode will have a solution of the form

(27) $$\begin{array}{c} h_{n}\left(t\right)=h_{n}^{\left(h\right)}\left(t\right)+h_{n}^{\left(i\right)}\left(t\right) \end{array}$$

where $h_{n}^{\left(h\right)}$ is the homogeneous solution and $h_{n}^{\left(i\right)}$ is the inhomogeneous solution for the forced wavemode propagation initialized from zero initial conditions. The general form of the former can be used to characterize the decay transience

(28) $$\begin{array}{c} h_{n}^{\left(h\right)}\left(t\right)=a_{+,n}^{\left(h\right)}\;e^{r_{+,n}\;t}+a_{-,n}^{\left(h\right)}\;e^{r_{-,n}\;t} \end{array}$$

where the coefficients $a_{\pm ,n}^{\left(h\right)}$ are determined by the initial conditions and *r*_±, *n*_ are the eigenvalues of the left side of Equation (11) (the roots of the characteristic equation) as

(29) $$\begin{array}{c} r_{\pm ,n}=-\frac{1}{2}\left(b_{1,n}\pm \sqrt{b_{1,n}^{2}-4\;b_{0,n}}\right) \end{array}$$

Then the discriminant determines the character of the wavemode

(30) $$\begin{array}{c} b_{1,n}^{2}-4\;b_{0,n}\;\left\{\begin{array}{c} >0,\;r_{\pm ,n}\in R,\;\text{two}\;\text{distinct}\;\text{roots}\\ =0,\;r_{\pm ,n}\in R,\;\text{two}\;\text{degenerate}\;\text{roots}\\ <0,\;r_{\pm ,n}\in C,\;\text{two}\;\text{conjugate}\;\text{roots} \end{array}\right. \end{array}$$

The physical conditions for degeneracy required an exacting degree of marginality rarely (if ever) encountered in real systems, so that this solution type may be safely ignored (degeneracy corresponds to algebraic growth at small times that was mediated by exponential decay at longer times).

Rewriting the conditions (30) in terms of physical parameters, it was found that

(31) $$\begin{array}{c} n\;\left\{\begin{array}{c} >\sqrt{\frac{2}{\pi }}\;Oh^{-1},\;r_{\pm ,n}\in R,\;\text{strictly}\;\text{decaying}\;\mathrm{wavemode}\\ <\sqrt{\frac{2}{\pi }}\;Oh^{-1},\;r_{\pm ,n}\in C,\;\text{oscillatory}\;\text{decaying}\;\mathrm{wavemode} \end{array}\right. \end{array}$$

where

(32) $$\begin{array}{c} Oh=\frac{\eta }{\sqrt{\rho \;\gamma \;d}} \end{array}$$

is the Ohnesorge number characterizing the balance between the dissipative viscous effects and the conservative effects resulting from interaction between inertia and surface tension. There exists a condition for oscillation of the unforced membrane and this condition is $Oh<\sqrt{2/\pi }$. When $Oh\geq \sqrt{2/\pi }$, no oscillatory unforced wavemodes are permitted and the unforced membrane will not oscillate. When the condition is satisfied, it was observed that oscillation can be attributed exclusively to wavemodes with the “smallest” mode numbers, and that these will always include the fundamental mode. Figure S3, Supporting Information represents the change in *Oh* for a range of surface tensions and membrane length.

### Ultrasound Transducer Fabrication

A set of custom‐made single crystalline 127.86 Y‐rotated X‐propagating lithium niobate transducers operating in the thickness mode were used, as previously described.^[^ ^73^ ^]^ The fundamental frequency was measured to be 6.72 MHz using noncontact laser Doppler vibrometry (UHF‐120SV, Polytec, Waldbronn, Germany). The transducers were coated with a 1 μm layer of Au atop 20 nm of Ti acting as an adhesion layer, using a direct‐current sputtering (Denton 635 DC Sputtering system) process was used to coat 4 inch diameter wafers in an inert gas environment with a 2.3 mTorr pressure and rotation speed of 13 rpm, at a deposition rate of 1.5 A s^−1^ for Ti and 7 A s^−1^ for Au. Devices were diced to size (12 mm × 12 mm) and built in to the in vitro test setup using an automated dicing saw (DISCO 3220, DISCO, Tokyo Japan).

### Rat Primary Neuron Culture

Rat primary neuronal cultures were prepared from rat pup tissue at embryonic days (E) 18 containing combined cortex, hippocampus, and ventricular zone. The tissue was obtained from BrainBits (Catalog #: SDEHCV) in Hibernate‐E media and used the same day for dissociation following their protocol.

Briefly, tissue was incubated in a solution of papain (BrainBits PAP) at 2 mg mL^−`^ for 30 min at 37 °C and dissociated in Hibernate‐E for 1 min using one sterile 9” silanized Pasteur pipette with a fire‐polished tip. The cell dispersion solution was centrifuged at 1100 rpm for 1 min, and the pellet was resuspended with 1 mL NbActiv1 (BrainBits NbActiv1 500 mL). The cell concentration was determined using a haemocytometer (TC20, Bio‐Rad Labs, Hercules, California, USA) and neurons were plated in 12‐well culture plates with 18‐mm PDL‐coated coverslips (GG‐18‐PDL, Neuvitro Corporation, Vancouver, Washington, USA) at a concentration of 1.3 million cells per well. Neurons were then incubated at 37 °C, 5% CO_2_, performing half media changes every 3–4 days with fresh NbActiv1 supplemented with PrimocinTM (ant‐pm‐1, InvivoGen, San Diego, California, USA). Cultures were incubated at 37 °C, 5% CO_2_ until day 10–12 and were used in DHM imaging experiments.

### In‐Vitro Electrophysiology

A stable line of neurons using the protocol listed above were cultured on 18 mm round coverslips, at a seeding density of ≈300 k cells per well in a tissue‐culture treated 12‐well plate. Neurons were allowed to mature for 11–14 days in vitro prior to recording. Coverslips were transferred to a custom machined acrylic stage containing a bath of external solution; NaCl (140 mm), KCl (4 mm), MgCl_2_ (2 mm), glucose (5 mm), and HEPES (10 mm) with an osmolarity of ≈290 mOsm. Patch pipettes were pulled on a pipette puller (P‐97, Sutter Instruments, Novato, CA, USA) programmed to give 4–6 MΩ tips from filamented borosilicate glass (o.d. 1.5 mm, i.d. 0.86 mm) and used with an internal solution comprising of a CsF and KF base (#08 3008 and #08 3007, respectively, Nanion, Munich, Germany). A 40× water dipping lens (LUMPLFLN40XW, Olympus Corporation, Tokyo, Japan) with a numerical aperture (NA) of 0.8 was used in combination with a complementary metal oxide semiconductor camera (01‐OPTIMOS‐R‐M‐16‐C QImaging OptiMOS, Roper Technologies, USA) to visualize cells with Köhler or fluorescent illumination. Electrical signals were acquired using an amplifier (Axon Instruments Multiclamp 700B, Molecular Devices LLC, California, USA) and digitized (Axon Instruments Digidata 1550B, Molecular Devices LLC, California, USA) using an acquisition and control software (pClamp 11, Molecular Devices LLC, California, USA). Gap free recordings were conducted (typically holding the membrane potential at −70 mV) while delivering the ultrasound stimulus. The ultrasound delivery rig used for patch clamp experiments was the same used for imaging experiments. Briefly, waveforms were programmed using an arbitrary function generator (33600A Series, Keysight, California, USA) connected via BNC to an amplifier (TC2057574, Vox Technologies, Richardson, TX). Military communications grade BNC (Bayonet Neill–Concelman) cables (CA5512‐36, Federal Custom Cable, California, USA) were used to ensure impedance matching in the systems and reduce electrical interference. The amplifier was connected to the custom‐made lithium niobate transducer mounted on a dovetail sliding arm, and coupled to the bottom of the recording chamber with ultrasound gel. Recordings were carried out in response to peak pressures of 0.5 MPa as access resistance could not be maintained when high pressures were delivered. Upon successful whole‐cell access, baseline gap‐free recordings in current clamp trials were obtained. Access resistance during successful whole‐cell recordings was maintained between 10 to 25 MΩ.

### Statistical Analysis

The reconstructed holograms from the digital holographic microscope was exported using Koala (Lynceé‐tec Inc., Lausanne, Switzerland) and analyzed using custom code written using MATLAB (Mathworks, Natlic, MA, USA) and ImageJ (National Institutes of Health, Bethesda, MD, USA). Line profiles along the length of the cell were exported using ImageJ for every frame and the mean baseline profile was calculated for each cell. The maximum deflection during the applied stimulus was then calculated for each cell by comparing the profile during the stimulus to the mean profile before the stimulus. Figure 2c represents the maximum deflections of each neuron and HEK cell from the baseline during the applied stimulus. Figure 2d–f represent the mean and maximum deflections when there was no ultrasound (green) and when the ultrasound stimulus was delivered (red).

## Conflict of Interest

The authors declare no conflict of interest.

## Author Contributions

A.V., S.C., and J.F. designed the experiments. A.V. conducted the experiments. A.V. developed the model with support from J.O. and J.F. U.M. and C.W. performed the HEK cell culture. M.D. and A.V. performed the primary neuron culture. Y.T. performed the electrophysiology. A.V. wrote the paper with edits from J.F., J.O., and S.C.

## Supporting information

Supporting Information

Supplemental Video 1

Supplemental Video 2

## Data Availability

The data that support the findings of this study are available from the corresponding author upon reasonable request.
