# Supplementary material for: Ultrasound Mediated Cellular Deflection Results in Cellular Depolarization
Source: Adv Sci (Weinh). 2021 Nov 7;9(2):2101950. doi: 10.1002/advs.202101950 (PMC8805560; doi:10.1002/advs.202101950)
Supplement: Supplementary file 1 — Supporting Information [file ADVS-9-2101950-s001.pdf]

Ultrasound mediated cellular deflection results in cellular depolarization

*Aditya Vasan Jeremy Orosco Uri Magaram Marc Duque Connor Weiss Yusuf Tufail Sreekanth H Chalasani James Friend**

Aditya Vasan, Jeremy Orosco, James Friend
Medically Advanced Devices Laboratory, Department of Mechanical and Aerospace Engineering, Jacobs School of Engineering and Department of Surgery, School of Medicine, University of California San Diego, La Jolla CA 92093 USA
Uri Magaram, Marc Duque, Connor Weiss, Yusuf Tufail, Sreekanth H Chalasani
Molecular Neurobiology Laboratory, The Salk Institute for Biological Studies, La Jolla, CA 92037

Email address: jfriend@ucsd.edu
Webpage: http://friend.ucsd.edu

Keywords: *ultrasound, neuromodulation, digital holographic microscopy, acoustofluidics*

Supplementary Videos

Video S1 | HEK cell membrane deflection when subjected to an ultrasound stimulus
Representative response to a 50 ms ultrasound burst. The video duration is 100 ms with an initial 25 ms baseline recording. The transducer has an operating frequency of 6.72 MHz.
Video S2 | Neuron membrane deflection when subjected to an ultrasound stimulus
Representative response to a 50 ms ultrasound burst. The video duration is 100 ms with an initial 25 ms baseline recording. The transducer has an operating frequency of 6.72 MHz.

Whole cell current clamp electrophysiology: additional results

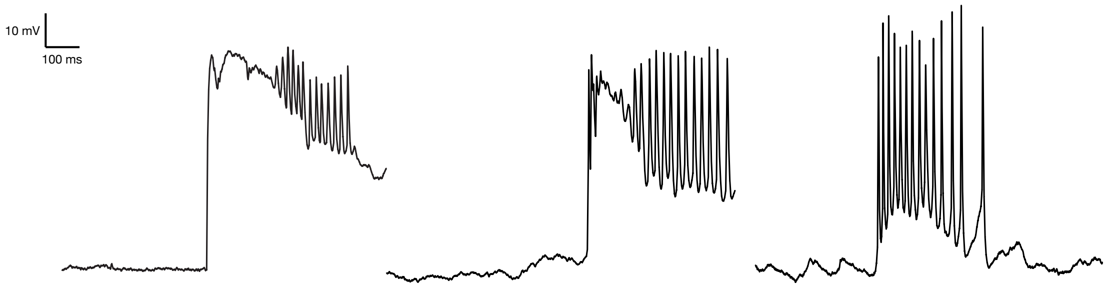

Figure S1: Current clamp electrophysiology data on neurons subjected to ultrasound stimuli.

Whole cell current clamp electrophysiology performed on rat primary neurons shows rapid oscillations of voltage corresponding to the onset of the ultrasound stimulus (Fig. S1).

Centered-difference scheme for deflection verification

A centered difference scheme may also be employed for discretizing the temporal and spatial derivatives of the partial differential equation governing membrane deflection:

$$\frac{\partial^2 u}{\partial t^2} = \frac{u_i^{n+1} - 2u_i^n + u_i^{n-1}}{h^2}, \tag{S.1a}$$

$$\frac{\partial^2 u}{\partial x^2} = \frac{u_{i+1}^n - 2u_i^n + u_{i-1}^n}{k^2}, \tag{S.1b}$$

$$\frac{\partial^3 u_z}{\partial x^2 \partial t} = \frac{u_{i+1}^n - u_{i+1}^{n-1} - 2(u_i^n - u_i^{n-1}) + u_i^n - u_{i-1}^{n-1}}{hk^2}. \tag{S.1c}$$

The displacement of the membrane may be determined by iterating with these finite difference equations.

Deflection due to cavitation-based models

Our model predicts deflection due to ultrasound and in later sections, we demonstrate how deflection results in the generation of action potentials. This is in contrast to other proposed mechanisms for cellular activity mediated by ultrasound, for example, by cavitation [1]. Cavitation may either be inertial, where there is rapid collapse, or stable, where there are steady pulsations. Given the range of ultrasound parameters that may induce a neuronal response, there are major limitations with a cavitation based approach for explaining the observed bioeffects as cavitation is less likely to occur at higher frequencies of operation. In addition to the particular frequency used for stimulation, another important factor is the nature of the waveform of acoustic excitation, as highlighted by Apfel *et al.* [2]. The range of pressures used for ultrasound stimulation are well below cavitation thresholds in the media of interest: tissue. This is critical as the displacement of the membrane at sub-cavitation pressures may be approximated by the perturbation in the fluid due to the ultrasound field alone.

In order to investigate any potential cavitation-based phenomenon, we considered the Rayleigh-Plesset equation and simulated the oscillation of a nanometer scale bubble. The smaller the bubble, the higher its resonance frequency and faster its response to an applied acoustic field. If the frequency of the applied field is less than the natural frequency of the bubble, the bubble may respond to tension in the fluid field thereby causing it to expand. When the field exerts a compressional event, the bubble collapses and results in a transient cavitation event. There are three possibilities: the bubble is large and has a slow response time to a compressional field, the bubble is very small and growth is inhibited by surface tension, or there is an intermediate size for the given acoustic field parameters that undergoes transient cavitation. The Blake threshold [3] establishes a lower limit for bubble radius, above which transient behavior is possible. This threshold is established by using the quasi-static assumption for pressure within the bubble and the liquid outside it,

$$p_L = \left(P_0 + \frac{2\sigma}{R_0}\right)\left(\frac{R_0}{R}\right)^{3\kappa} - \frac{2\sigma}{R} \tag{S.2}$$

where $\kappa = 1$ for the isothermal case. Differentiating the above with respect to R establishes the lower limit for transient cavitation. Assuming a 5 nm bubble, the critical radius is found to be 6.23 nm. Neppiras and Noltingk [4] established an upper threshold for bubble size, corresponding to a particular frequency above which transient cavitation conditions are not realized. This is given by

$$\rho\omega^2 R_0^2 = 3\gamma\left(P_a + \frac{2\sigma}{R_0}\right) - \frac{2\sigma}{R_0} \tag{S.3}$$

where ρ is the liquid density and γ is the ratio of specific heats for the gas within the bubble. Assuming a frequency of 7 MHz, the upper threshold is found to be 0.1 μm. One of the theories for ultrasound mediated neuromodulation [1] suggests a cavitation based phenomenon governing the generation of action potentials due to stable cavitation of dissolved gas in between the lipid bilayer. The lipid bilayer is approximately

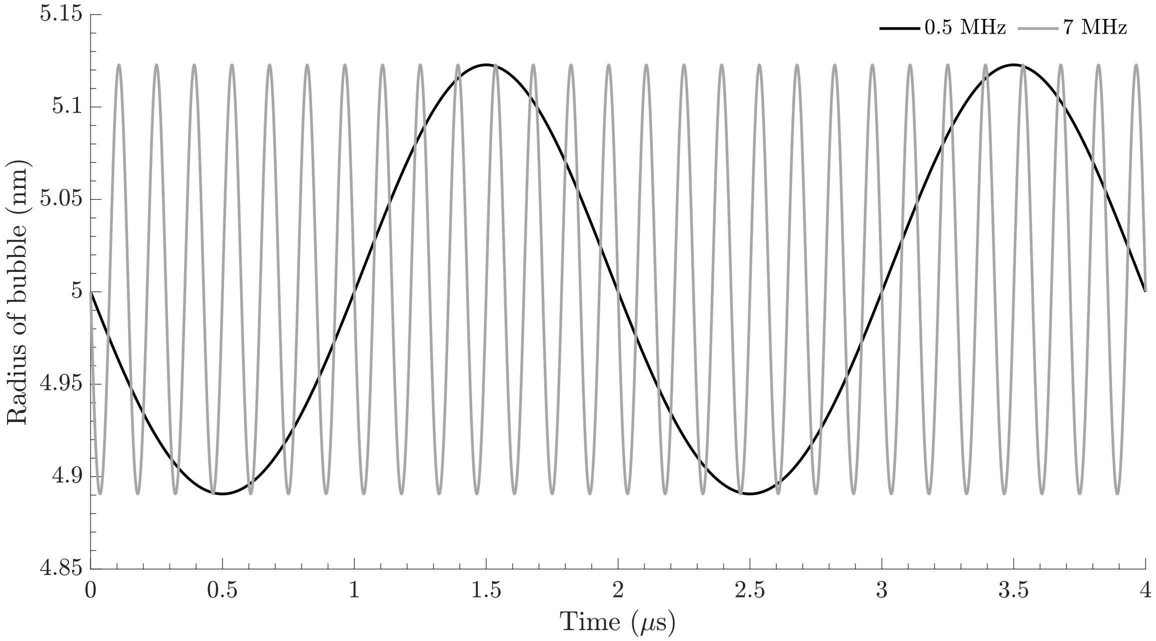

Figure S2: Sub nanometer oscillations of a bubble due to pressures at the mechanical index limit, showing that cavitation-based phenomenon are likely not responsible for generating membrane deflection.

7 nm thick [5] and considering the limits established with the above thresholds, stable cavitaion may be possible for a bubble enclosed within the leaflets. Forced oscillations of a bubble for small amplitudes is best described by the Rayleigh-Plesset equation,

$$\frac{d^2R}{dt^2} + \frac{3}{2R}\left(\frac{dR}{dt}\right)^2 = \frac{1}{\rho}\left\{\left(P_0 + \frac{2\sigma}{R_0} - P_v\right)\left(\frac{R_0}{R}\right)^{3\kappa} + P_v - \frac{2\sigma}{R} - \frac{4\eta}{R}\frac{dR}{dt} - P_0 - P(t)\right\} \tag{S.4}$$

where η is the viscosity of the surrounding fluid and $P(t)$ is the sinusoidal pressure variation due to the ultrasound field. Applying the Rayleigh-Plesset equation to a bubble with diameter on the order of magnitude of the thickness of the bilayer results in deflections on the order of ± 0.08 nm.

For a bubble diameter of 5 nm under the influence of a 1 MPa sinusoidal pressure field, the maximum change in radius is ± 0.08 nm from the initial diameter, comparable to Brownian thermal motion of lipids in the bilayer [**?**] This holds true even at lower stimulation frequencies, for example 0.5 MHz at a pressure of 1.35 MPa (corresponding to an MI of 1.9) results in a deflection of ± 0.1 nm.

References

[1] M. Plaksin, S. Shoham, and E. Kimmel, "Intramembrane cavitation as a predictive bio-piezoelectric mechanism for ultrasonic brain stimulation," *Physical Review X*, vol. 4, no. 1, p. 011004, 2014.

[2] R. E. Apfel and C. K. Holland, "Gauging the likelihood of cavitation from short-pulse, low-duty cycle diagnostic ultrasound," *Ultrasound in Medicine & Biology*, vol. 17, no. 2, pp. 179–185, 1991.

[3] F. Blake, "Technical memo 12," *Acoustics Research Laboratory, Harvard University, Cambridge, MA*, 1949.

[4] B. E. Noltingk and E. A. Neppiras, "Cavitation produced by ultrasonics," *Proceedings of the Physical Society. Section B*, vol. 63, no. 9, p. 674, 1950.

[5] B. A. Lewis and D. M. Engelman, "Lipid bilayer thickness varies linearly with acyl chain length in fluid phosphatidylcholine vesicles," *Journal of Molecular Biology*, vol. 166, no. 2, pp. 211–217, 1983.

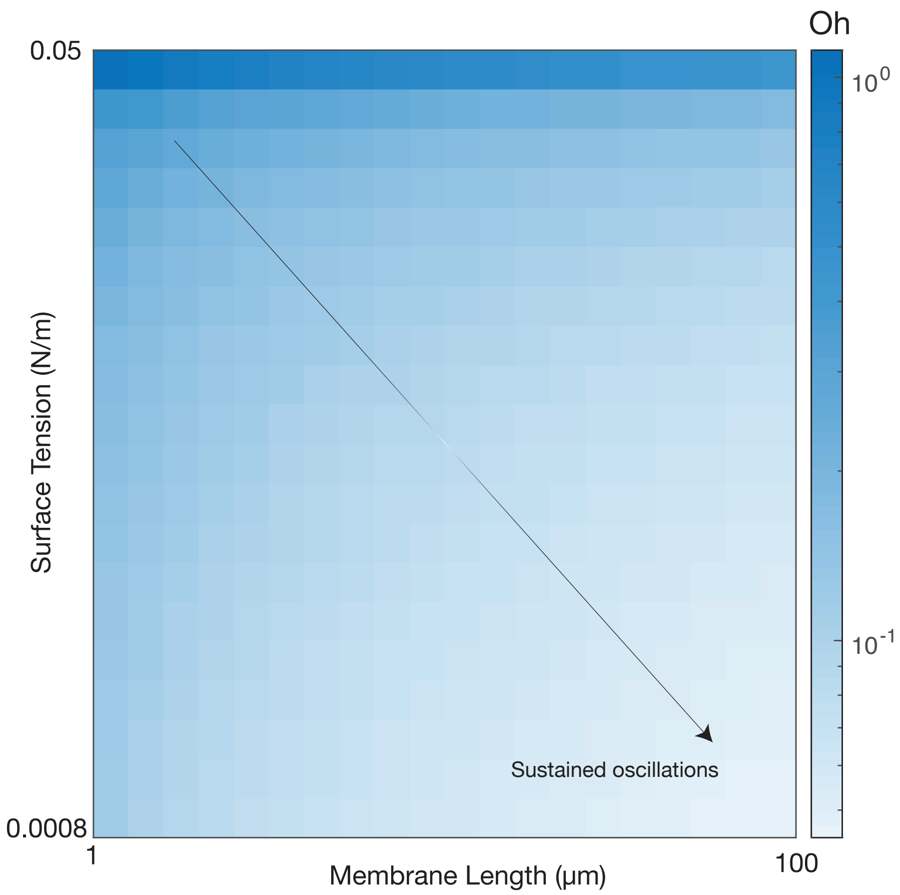

Figure S3: The Oh number relates viscous forces to inertial and surface tension forces. In this case, the viscosity and the density are assumed constant. Increased forces result in damped oscillation on the membrane. Changes in surface tension and membrane length determine if the membrane may sustain oscillations.
